# Recent Advances in Complex Networks Theories with Applications

**DOI:** 10.1155/2014/504923

**Published:** 2014-07-23

**Authors:** Hamid Reza Karimi, Wei Zhang, Xuebo Yang, Zhandong Yu

**Affiliations:** ^1^Department of Engineering, Faculty of Engineering and Science, University of Agder, 4898 Grimstad, Norway; ^2^Department of Management Science and Engineering, School of Management, Harbin Institute of Technology, Harbin, China; ^3^Research Institute of Mechatronics and Automation, Bohai University, Jinzhou, Liaoning, China; ^4^College of Automation, Harbin Engineering University, Harbin, China

Since the 1980s, the rapid development of computer and information engineering technology represented by the Internet makes the human society stride into the “network era.” From the Internet to the World Wide Web, from the power grid to the transportation network, from the organism's brain neural networks to the metabolic network, and from research cooperation network to a variety of political, economic, and social networks, people have actually lived in a world filled with a wide variety of complex networks. Hence, in recent years, complex networks have become the research focus in different disciplines including mechanical, physics, biology, system control, communication technology, social, economic, and military disciplines. Previous studies have mainly focused on the following: (1) static statistical analysis of complex network topology, such as the matching pattern based on a given degree distribution, a variety of correlation, and the description of weighted networks; (2) the evolution and mechanisms model of complex networks, such as network evolution statistical regularity and geometric properties of network; (3) kinetics of complex networks, including network fault tolerance and attack robustness, and the dynamic processes of spread, synchronization, and resonance on the network.

The overall aim of this special issue is to bring together the latest/innovative knowledge and advances in theoretical and technological developments and their applications to complex networks, which may depend largely on methods from artificial intelligence, statistics, operational research, management science, and engineering.

This special issue contains twenty-eight papers, the contents of which are summarized as follows.

“*Extraction of multilayered social networks from activity data*” by K. Musial et al. proposes a new method for creation of multilayered social network based on the data about users activities towards different types of objects between which the hierarchy exists. Due to the flattening, preprocessing procedure new layers and new relationships in the multilayered social network can be identified and analysed.

“*A social diffusion model with an application on election simulation*” by J.-K. Lou et al. designs a diffusion model which is capable of managing the aforementioned scenario. To demonstrate the usefulness of our model, the authors simulate the diffusion on the network built based on a publicly available bibliography dataset.

“*An energy efficient simultaneous-node repositioning algorithm for mobile sensor networks*” by M. A. Khan et al. addresses the concerns of both connectivity and coverage in an integrated way so that this gap can be filled. A novel algorithm for simultaneous-node repositioning is introduced. In this approach, each neighbor of the failed node, one by one, moves in for a certain amount of time to take the place of the failed node, after which it returns to its original location in the network. The effectiveness of this algorithm has been verified by the simulation results.

“*Joint estimation of 2D-DOA and frequency based on space-time matrix and conformal array*” by L.-T. Wan et al. studies a joint frequency and two-dimension DOA (2D-DOA) estimation algorithm for conformal array. The delay correlation function is used to suppress noise. Both spatial and time samplings are utilized to construct the spatial-time matrix. The frequency and 2D-DOA estimation are accomplished based on parallel factor (PARAFAC) analysis without spectral peak searching and parameter pairing. The proposed algorithm needs only four guiding elements with precise positions to estimate frequency and 2D-DOA. Other instrumental elements can be arranged flexibly on the surface of the carrier. Simulation results demonstrate the effectiveness of the proposed algorithm.

“*Beamforming of joint polarization-space matched filtering for conformal array*” by L. Liu et al. investigates a novel joint polarization-space matched filtering algorithm for cylindrical conformal array. First, the snapshot data model of the conformal polarization sensitive array is analyzed. Second, the analytical expression of polarization sensitive array beamforming is derived. Linearly constrained minimum variance (LCMV) beamforming technique is facilitated for the cylindrical conformal array. Third, the idea of joint polarization-space matched filtering is presented, and the principle of joint polarization-space matched filtering is discussed in detail.

“*The robustness analysis of wireless sensor networks under uncertain interference*” by C. Deng presents robustness analysis of condition monitoring wireless sensor network under uncertain interference based on the complex network theory. In the evolution of the topology of sensor networks, the density weighted algebraic connectivity is taken into account, and the phenomenon of removing and repairing the link and node in the network is discussed. Numerical simulation is conducted to explore algebraic connectivity characteristics and network robustness performance. It is found that nodes density has an effect on algebraic connectivity distribution in the random graph model; high density nodes carry more connections, use more throughputs, and may be more unreliable.

“*Reconfiguration and search of social networks*” by L. Zhang et al. presents a new network model which suits to portray the structure of social networks based on the topological characteristics of the real social networks, and the characteristic parameters of the model were calculated. To find out the relationship between two people in the social network, and using the local information of the social network and the parallel mechanism, a hybrid search strategy based on walker random and a high degree was proposed. Simulation results show that the strategy can significantly reduce the average number of search steps, so as to effectively improve the search speed and efficiency.

“*A new cooperative MIMO scheme based on SM for energy-efficiency improvement in wireless sensor network*” by Y. Peng and J. Choi studies a new cooperative multiple-input multiple-output (CMIMO) scheme based on the spatial modulation (SM) technique named CMIMO-SM for energy-efficiency improvement. They first establish the system model of CMIMO-SM. Based on this model; the transmission approach is introduced graphically. In order to evaluate the performance of the proposed scheme, a detailed analysis in terms of energy consumption per bit of the proposed scheme compared with the conventional CMIMO is presented. Later, under the guide of this new scheme they extend their proposed CMIMO-SM to a multihop clustered WSN for further achieving energy efficiency by finding an optimal hop length.

“*Augmented Lagrange based on modified covariance matching criterion method for DOA estimation in compressed sensing*” by W. Si et al. addresses a novel direction of arrival (DOA) estimation method in compressed sensing (CS), in which DOA estimation is considered as the joint sparse recovery from multiple measurement vectors (MMV). The proposed method is obtained by minimizing the modified-based covariance matching criterion, which is acquired by adding penalties according to the regularization method. This minimization problem is shown to be a semidefinite program (SDP) and transformed into a constrained quadratic programming problem for reducing computational complexity which can be solved by the augmented Lagrange method. The proposed method can significantly improve the performance especially in the scenarios with low signal to noise ratio (SNR), small number of snapshots, and closely spaced correlated sources.

“*Channel selection based on trust and multiarmed bandit in multiuser, multichannel cognitive radio networks*” by F. Zeng and X. Shen presents a channel selection scheme for the multiuser, multichannel cognitive radio networks. This scheme formulates the channel selection as the multiarmed bandit problem, where cognitive radio users are compared to the players and channels to the arms. By simulation negotiation we can achieve the potential reward on each channel after it is selected for transmission; then the channel with the maximum accumulated rewards is formally chosen.

“*Energy-efficient routing control algorithm in large-scale WSN for water environment monitoring with application to Three Gorges Reservoir area*” by Y. Zhong et al. studies a new energy-saving routing algorithm to maximally prolong lifetime of large-scale WSN, using the method of maximum energy-welfare optimization clustering. Firstly, temporary clusters are formed based on two main parameters, the remaining energy of nodes and the distance between a node and the base station. Secondly, the algorithm adjusts cluster heads and optimizes the clustering according to the maximum energy welfare of the cluster by the cluster head shifting mechanism. Finally, in order to save node energy efficiently, cluster heads transmit data to the base station in single-hop and multihop way.

“*Distributed leader-following finite-time consensus control for linear multiagent systems under switching topology*” by X. Xu et al. investigates the finite-time consensus problem of leader-following multiagent systems. The authors propose a sufficient condition to design the observer-based consensus protocol, which makes the multiagent systems achieve finite-time consensus under switching topologies.

“*Content patterns in topic-based overlapping communities*” by S. A. Ríos and R. Muñoz studies the problem of understanding the underlying community structure for social network analysis. The authors present a hybrid algorithm which combines two different overlapping subcommunity detection approaches and a method to analyze and compare the content generated.

“*In-band asymmetry compensation for accurate time/phase transport over optical transport network*” by S. Siu et al. studies the issues that are relevant to distributing accurate time/phase over optical transport network (OTN). With the proposed scheme in this paper, the fiber link delay asymmetry can be compensated relying on the estimated mean fiber link delay by the Telecom-Boundary clock, while the OTN control plane is responsible for processing the fiber link delay asymmetry to determine the asymmetry compensation in the timing chain.

“*Modeling of information diffusion in Twitter-like social networks under information overload*” by P. Li et al. takes Twitter-like social networks into account and proposes models to characterize the process of information diffusion under information overload. The results are of importance to understand the diffusion dynamics in social networks, and this analysis framework can be extended to consider more realistic situations.

“*Secure and fair cluster head selection protocol for enhancing security in mobile ad hoc networks*” by B. Paramasivan and M. Kaliappan studies the problem of secure and fair cluster head selection for mobile ad hoc networks (MANETs). The authors present a secure and fair cluster head selection protocol (SFCP) which integrates security factors into the clustering approach for achieving attacker identification and classification.

“*SVM-based spectrum mobility prediction scheme in mobile cognitive radio networks*” by Y. Wang et al. studies the problem of spectrum mobility which has not been fully investigated in mobile cognitive radio networks (CRNs). The authors propose a novel support vector machine based spectrum mobility prediction (SVM-SMP) scheme by considering time-varying and space-varying characteristics simultaneously in mobile CRNs.

“*A fast overlapping community detection algorithm with self-correcting ability*” by L. Cui et al. defines a weighted modularity based on the density and cohesion as the new evaluation measurement due to the defects of all kinds of modularity. The authors propose three test conditions for overlapping nodes and present a fast overlapping community detection algorithm with self-correcting ability, which is decomposed into two processes. And they also give a new understanding on membership vector.

“*Motion adaptive vertical handoff in cellular/WLAN heterogeneous wireless network*” by L. Li et al. studies the problem of vertical handoff for guaranteeing quality of service and overall performance of network. The authors propose a vertical handoff algorithm based on Q-learning. Meanwhile, neural fuzzy inference system (NFIS) is embedded to retain a continuous perception of the state space.

“*An improved proportionate normalized least-mean-square algorithm for broadband multipath channel estimation*” by Y. Li and M. Hamamura makes use of the sparsity property of broadband multipath wireless communication channels. The authors mathematically propose an lp-norm-constrained proportionate normalized least-mean-square (LP-PNLMS) sparse channel estimation algorithm which can effectively improve the estimation performance of the PNLMS-based algorithm for sparse channel estimation applications.

“*A novel complex networks clustering algorithm based on the core influence of nodes*” by C. Tong et al. investigates the network clustering problem which is significant for the study of complex networks. The authors propose a clustering algorithm by calculating the core influence of nodes. The clustering accuracy of this algorithm is superior to the classical clustering algorithm (fast Newman algorithm). It clusters faster and plays a positive role in revealing the real cluster structure of complex networks precisely.

“*Robust H*_*∞*_ *filtering for a class of complex networks with stochastic packet dropouts and time delays*” by J. Zhang et al. studies the problem of robust *H*_*∞*_ filtering for a class of complex network systems which has stochastic packet dropouts and time delays, combined with disturbance inputs. The authors aim to design a filter such that the estimation error converges to zero exponentially in the mean square, while the disturbance rejection attenuation is constrained to a given level by means of the performance index.

“*Discovering the influences of complex network effects on recovering large scale multiagent systems*” by Y. Xu et al. presents an initial effort to find how a standard network recovery policy, MPLS algorithm, may change the network topology of the multiagent system in terms of network congestion. The authors have established that when the multiagent system is organized as different network topologies according to different complex network attributes, the network shifts in different ways.

“*WDM network and multicasting protocol atrategies*” by P. Kirci and A. H. Zaim studies a new multicasting protocol with optical burst switching (OBS). The authors examine the performance of the protocol with just enough time (JET) and just in time (JIT) reservation protocols. Also, the paper involves most of the recent advances about wavelength division multiplexing (WDM) multicasting in optical networks.

“*Assessment on knowledge network sharing capability of industrial cluster Based on Dempster-Shafer theory of evidence*” by S. Dai and H. Zhang presents a concept model of knowledge sharing network based on theory of evidence. Next, the authors create a set of assessment index systems. The research result shows relatively high knowledge network sharing capacity among the certain industrial cluster firms.

“*A novel joint problem of routing, scheduling, and variable-width channel allocation in WMNs*” by C.-C. Lin et al. studies a novel joint problem of routing, scheduling, and channel allocation for single-radio multichannel wireless mesh networks. As a result, this paper first constructs a linear programming model with more practical concerns and then proposes a simulated annealing approach with a novel encoding mechanism.

“*Modeling and simulation of complex network attributes on coordinating large multiagent system*” by Y. Xu et al. presents simulation testbed CoordSim built to model the coordination of network centric multiagent systems. The authors have theoretically analyzed that the characters of complex network make a significant difference with both random and intelligent coordination strategies.

“*Towards an optimal energy consumption for unattended mobile sensor networks through autonomous sensor redeployment*” by J. Chen et al. studies an autonomous sensor redeployment algorithm to balance energy consumption and mitigate energy hole for unattended mobile sensor networks.

Of course, the selected topics and papers are not a comprehensive representation of the area of this special issue. Nonetheless, they represent the rich and many-faceted knowledge that we have the pleasure of sharing with the readers.

